# Chemical messages from an ancient buried bottle: metabolomics for wine archeochemistry

**DOI:** 10.1038/s41538-017-0001-5

**Published:** 2017-10-30

**Authors:** Chloé Roullier-Gall, Silke S. Heinzmann, Jean-Pierre Garcia, Philippe Schmitt-Kopplin, Régis D. Gougeon

**Affiliations:** 1 Technische Universitat Munchen, Chair of Analytical Food Chemistry, Alte Akademie 10, 85354 Freising-Weihenstephan, Germany; 2 German Research Center for Environmental Health, Research Unit Analytical BioGeoChemistry, Helmholtz Zentrum München, Ingolstadter Landstrasse. 1, 85764 Neuherberg, Germany; 3 UMR 6298 ARTEHIS, Université de Bourgogne/CNRS/culture, Université de Bourgogne, 6 bd Gabriel, 21000 Dijon, France; 4 UMR A 02.102 PAM Université de Bourgogne/Agrosup Dijon, Equipe PCAV, Institut Universitaire de la Vigne et du Vin, Jules Guyot, Rue Claude Ladrey, BP 27877 Dijon Cedex, France

**Keywords:** Metabolomics, Secondary metabolism, Complexity

## Abstract

Restoration works in the old Clunisian Saint-Vivant monastery in Burgundy revealed an unidentified wine bottle (SV1) dating between 1772 and 1860. Chemical evidence for SV1 origin and nature are presented here using non-targeted Fourier Transform Ion Cyclotron Resonance Mass Spectrometry and Nuclear Magnetic Resonance analyses. The SV1 chemical diversity was compared to red wines (Pinot Noir) from the Romanée Saint Vivant appellation and from six different vintages spanning from 1915 to 2009. The close metabolomic signature between SV1 and Romanée Saint Vivant wines spoke in favor of a filiation between these wines, in particular considering the Pinot noir grape variety. A further statistical comparison with up to 77 Pinot noir wines from Burgundy and vintages from nearly all the 20th century, confirmed that SV1 must have been made more than one hundred years ago. The increasing number of detected high masses and of nitrogen containing compounds with the ageing of the wine was in accordance with known ageing mechanisms. Besides, resveratrol was shown here to be preserved for more than one hundred years in wine. For the first time, the age of an old unknown wine along with its grape variety have been assessed through non-targeted metabolomic analyses.

## WINE ARCHEOCHEMISTRY: Identify an ancient wine via metabolomics analyses

For the first time, non-targeted metabolomics analyses were employed to assess the age of an unlabeled old wine together with its grape variety. Roullier-Gall at the Technische Universitat Munchen and coworkers from both Germany and France identified the metabolomics of unknown wine SV1 using high resolution FT-ICR Mass Spectrometry in combination with NMR. By comparing the metabolic signature of SV1 with the reference wines, it was found the SV1 wine was more than 100 years old and its grape variety was close to the Pinot Noir from the Romanée Saint Vivant appellation. Moreover, this was the first time that resveratrol was observed in wines of more than one hundred years. This archeochemical method can serve as an alternative approach to the commonly used genetic-based techniques on the wine authentication.

## Introduction

If a wealth of analytical data has already been published about the composition of grape and wine,^1–5^ recent non targeted analyses have emphasized the extent of the yet unknown bio-chemistry involved throughout the winemaking process.^6–8^ Such quest for the description and the understanding of the composition of wine is undoubtedly fostered by its various features ranging from organoleptic to health-related properties.^9–11^ However, it is also driven by a unique universally shared cultural heritage, which has always aroused the curiosity of both scientists and non-scientists for this specific food product, “transformed to be consumed within a culturally constructed set of rules and beliefs, often in situations strongly associated with reinforcing social identity”.^12^ This excitement is all the more pronounced that the wine in question is aged, precisely because of the messages it may hold from the time—supposedly far away—when it has been made. To that respect, archaeochemistry is becoming increasingly powerful for assessing time-related features such as the revealing of the beginning of winemaking through the analysis of traces of tartaric acid^13,14^ or the long-lasting antioxidant properties of a 600-year old fermented fruit juice found in amphorae in the relict of a ship of the Sicilian coast.^15^ By comparing the DNA sequences of modern *Saccharomyces cerevisiae* with those present in residue from ancient wine jars, DNA traceability indicates that this organism was probably responsible for wine fermentation by 3150 BC at least.^16^

Extending targeted analytical approaches to combined modern non-targeted analyses now provides unprecedented chemical snapshots in terms of molecular resolution and chemical diversity, that can allows to read messages related to various aspects of the winemaking practices^17–19^ or describing more generally the chemistry of complex unknown wine in a wide range of bio-geo-chemical samples.^20–23^ In a recent paper, we have shown that multiple analytical tool approaches enabled us to characterize the composition of 170-year old Champagne samples found in a shipwreck in the Baltic Sea.^19^

Here we show that ultrahigh-resolution FT-ICR-MS combined with NMR analyses of the contents of an unlabeled bottle buried in a cellar of the old Clunisian Saint-Vivant monastery can reveal for the first time a likely filiation with Pinot noir wines from the Côte de Nuits appellation.

## Results

### Historical considerations, typological examination of the bottle and radiocarbon analysis of its content

**Fig. 1:**
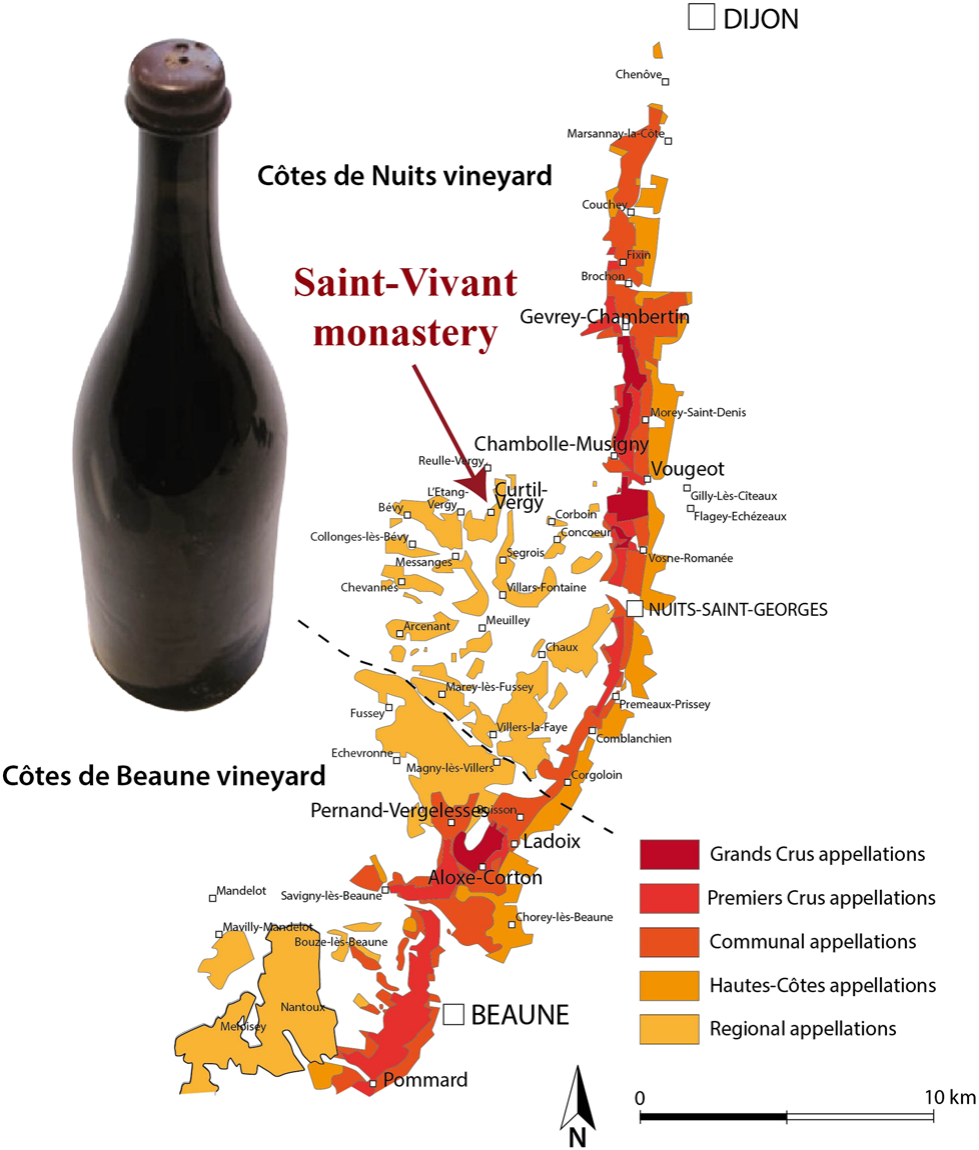
Map of the Saint-Vivant monastery location above Côte de Nuits vineyards (Burgundy, France) and picture of the SV1 bottle. Map generated using Adobe Illustrator CS6

The SV1 bottle was discovered and unearthed still full, buried 10 cm beneath a heavy limestone pavement remaining from the East wine cellar floor, in 2006 during the restoration works of the old Clunisian Saint-Vivant monastery (Fig. 1), the first owner of the most famous vineyards in Vosne-Romanée (Burgundy, France). The content of this bottle was finally sampled and analyzed in 2011. Historical records of this abbey indicate that it was built in ca. 900 AD, maintained in the Clunisian order during the Middle Ages and a modern reconstruction started in 1762 was achieved in 1772.^24^ However, it was almost completely destroyed during the French revolution in 1793, and only cellars were preserved until the late middle of the 19th century (24). Typologically, the SV1 75 cl wine bottle (Fig. 1) is of “Burgundy” style whose morphology is referred to, as those figured in Van den Bossche^25^ from France and Belgium during the 1780–1820 period (Supp. Fig. 1).

Radiocarbon analysis of organic deposits found at the bottle bottom (Miami Beta Analytic Inc laboratory sample Beta—310588) gave an analytic age of 90 ± 30 years BP and calibrated date ranges (95% probability) from either Cal AD 1680 to 1730 or Cal AD 1810 to 1930 (Supp. Fig. 1).

### 1D-^1^H and 2D ^1^H-^1^H TOCSY NMR analyses provide relative abundance of some known metabolites

In order to characterize the main features of the SV1 wine and to generate a direct comparison in terms of relative amounts of certain metabolites, we analyzed a contemporary Romanée Saint Vivant (RSV) wine from 2007, a nearly one hundred year old 1915 RSV wine and SV1, via 1D-^1^H NMR analyses (Supp. Fig. 2). Interestingly, the overall wine matrix was preserved in all three wines, as evidenced by the detection of alcohols, including ethanol, glycerol, 2,3-butanediol and isopentanol, or acids, with tartrate and lactate in particular (Table 1). Such results indicated higher concentrations of acids (*eg*. tartrate, lactate, formate, acetate) and lower concentrations of other metabolites (e.g., ethanol, glycerol, succinate, gallic acid) for SV1. Other compounds such as amino acids exhibited higher relative concentrations for the 1915 wine (Table 1).

**Table 1:** Quantification of selected families of metabolites in contemporary RSV wines, old wines from 1915 and SV1 via non-targeted 1D-^1^H NMR analyses

	Alcohols					Acids					
	EtOH	MeOH	2,3-Butandiol	Glycerol	Isopentanol	Short fatty acids	Tartrate	Lactate	Formate	Succinate	Acetate
SV1	46635.474	128.871	491.448	1785.147	14.953	234.67	576.826	1406.251	9.888	116.973	1594.453
RSV 1915	56288.516	86.104	493.171	2070.256	10.481	163.231	137.428	1323.426	6.862	226.023	356.918
RSV 2007	74191.88	77.999	368.418	2215.028	28.804	210.091	58.748	569.715	0.156	254.372	335.426

	Amino acids					Others		
	Valine	Isoleucine	Alanine	Glutamine*	Glutamate*	GABA	Trigonelline	Gallic acid
SV1	7.396	2.975	48.719	20.5	40.197	15.516	1.045	0.563
RSV 1915	11.186	5.217	74.354	24.393	50.337	20.069	0.94	1.974
RSV 2007	6.365	2.588	53.61	11.913	35.222	30.347	1.132	6.403

Quantification was done relatively to the proline signal

*EtOH* ethanol, *MeOH* methanol, *GABA* γ-aminobutyric acid

*Signal overlaps with others

2D ^1^H-^1^H TOCSY NMR spectra of the same samples allowed to further compare molecular features related to wine ageing, through the identification of interconnected networks of ^1^H spin couplings. The focus on the aromatic region of the spectra, between 6 and 8.5 p.p.m. (Supp. Fig. 2) revealed a broad background in the 6.5–7.2 p.p.m. region corresponding to oligomeric/macromolecular structures, which were present in the young 2007 wine, but absent from the older 1915 and SV1 samples. However, common features could also be observed in this aromatic region, including for instance the presence of Tyrosine (6.9 p.p.m.) and Phenylalanine (7.3 p.p.m.) amino acids in similar concentrations in all three wines.

### ESI(-) FT-ICR-MS analyses provide chemical fingerprints of Romanée Saint Vivant wines

**Fig. 2:**
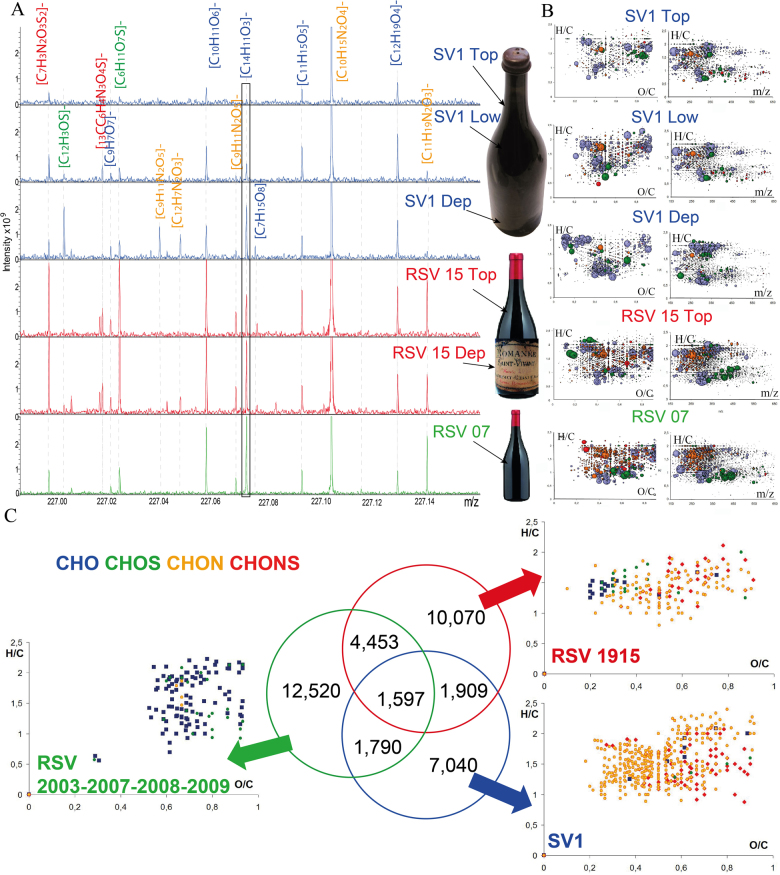
Negative-ion ESI FTICR mass spectra showing the overall similarity of Saint-Vivant wines. **a** Comparison of spectra in the *m*/*z* 226.90-227.20 Da mass range with the m/z 227.0713 identified by a blue box; **b** H/C vs. O/C van Krevelen diagrams and H/C vs. *m*/*z* diagrams of masses characteristic to SV1, RSV 1915, and RSV 2007 vintages. Bubble sizes indicate relative intensities of corresponding peaks in the spectra. **c** Venn diagram showing the counts of unique/common detected masses in the three following groups of wine: RSV 2003, 2007, 2008, 2009; RSV 1915 (Top and Dep); SV1 (Top, Low and Dep). Color code: CHO, blue; CHOS, green; CHON, red; CHONS, orange

Upon service, the SV1 wine exhibited a color intensity increase from top to bottom of the bottle. As a consequence, it was sampled at three levels (Fig. 2a), i.e., close to the above surface (Top), at roughly the middle of the largest body of the bottle (Low), and at the deposit level (Dep). To explore the chemical nature and generate an identity card, based on the comparison of non-targeted FT-ICR-MS signatures, these samples were analyzed by the previously described flow injection ESI(-) FT-ICR-MS,^20^ along with five supposedly related red wines, from the Saint-Vivant vineyard in Vosne Romanée, spanning vintages from 1915 to 2009, for comparison. Similarly to SV1, both the top part and the deposit of the 1915 wine were analyzed. Consistently with the observed color gradient in the bottle, the FT-ICR-MS analysis of SV1 samples actually revealed a striking gradient in both relative peak intensities and metabolic diversity from Top to Dep, as shown by van Krevelen diagrams (Fig. 2b). Various chemical families including carbohydrates, polyphenols, organic acids and fatty acids exhibited this trend towards an increased concentration from Top to Dep, with an overall higher abundance of CHO-based compounds in the deposit (Dep). Spectra enlargement at the m/z 227 nominal mass illustrated this chemical diversity gradient (Fig. 2a). Up to 16 major peaks were detected within this 400 mDa range, including in particular the peak at *m*/*z* 227.0713, corresponding to the [M − H]^−^ ion with [C_14_H_11_O_3_]^−^ absolute mass formula, most likely assignable to resveratrol isomers^26^ (Fig. 2a). Compositional differences, which exploit the exact mass information provided by FT-ICR-MS (24,25,28), further revealed that except for the N compositional difference, and to a lesser extent for the NH_2_ one, the number of possible compositional transformations was significantly higher for the 1915 and 2007 wines (Supp. Fig. 3). H_2_O or CH_2_O transformations showed a consistent decrease with the wine ageing, whereas NH, S or SO_3_ transformations appeared to be similar for the young 2007 wine and the nearly 100-year old 1915 wine (Supp. Fig. 3).

Considering all the masses detected (S/N 4) in spectra from the SV1 and RSV wines, the Venn diagram (Fig. 2c) showed that 12,520; 10,070 and 7040 unique masses could characterize the young wines, the RSV 1915 and the SV1 wine samples, respectively. Only 1597 of them were common to the three groups, although 60% (4453 + 1597) of the RSV 1915 specific masses were actually common to masses of the younger wines. In contrast, about 50% of the SV1 specific masses were common with those of the 1915 wine, or with those of the younger wines. Most interestingly, masses specific to 2003–2009 wines were mainly CHO compounds, whereas SV1 wine specific masses were characterized mostly by N-containing compounds (Fig. 2c). The RSV 1915 wine appeared as an intermediate, still exhibiting few CHO specific masses but mostly N-containing ones (Fig. 2c). A hierarchical cluster analysis (HCA) readily confirmed this progressive differentiation through the identification of the three groups (Supp. Fig. 4).

### Non-targeted metabolomics based on ESI(-) FT-ICR-MS analyses of wines from distinct grape variety and from a vertical series of Pinot noir wines

**Fig. 3:**
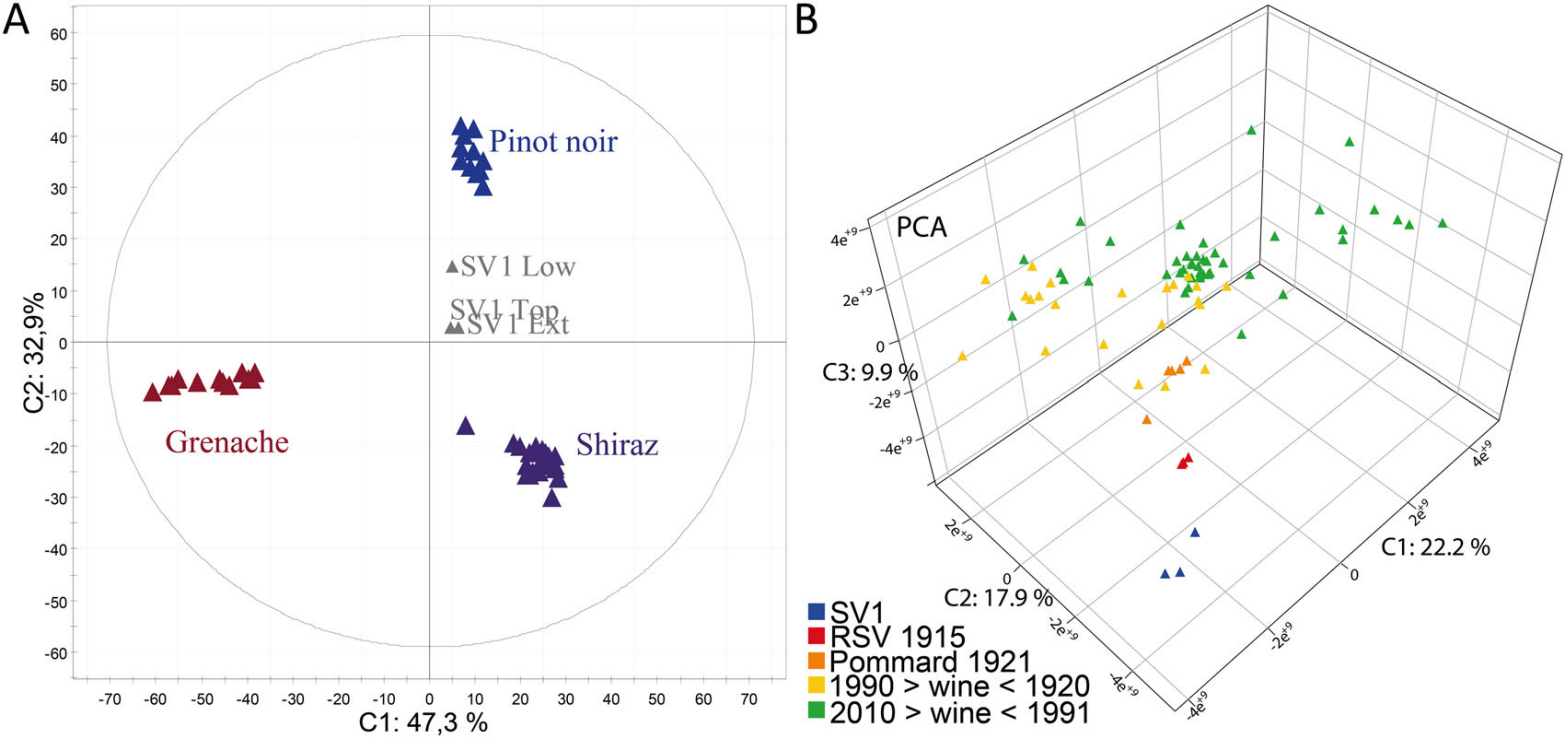
Multivariate statistical analyses of all mass features from FT-ICR-MS experiments. **a** PCA score plot of wines from the Tonnellerie 2000 experiment with three distinct grape varieties: Pinot noir (blue), Grenache (red) and Shiraz (purple), and showing SV1 wines (gray); PC1 and PC2 explained 47.3 and 32.9% of the variance. **b** PCA score plot of SV1 samples along with 77 Pinot noir red wines from Burgundy. PC1/PC2/PC3 explained 50.1% of the total variability (color code: wines younger than 1990, green; wine older than 1990, yellow; Pommard 1921 wines, orange; RSV 1915, red; SV1, blue)

In order to further reveal the likely Pinot noir identity of SV1, we used our previously published data from the Tonnellerie 2000 experiment,^26^ and in particular the set of discriminant masses for the three grape varieties involved (Pinot noir, Syrah and Grenache). The corresponding principal component analysis (PCA) score plot, including discriminant masses for SV1 samples (Top, Low, and Dep) illustrated the higher similarity between SV1 samples and Pinot noir wines (Fig. 3a). The two predictive components of the PCA (C1: 47.3% and C2: 32.9%) identified SV1 samples (Top, Low, and Dep) in the vicinity of Pinot noir wines.

A further indication of the evolution state of the SV1 wine was provided by an unsupervised PCA statistical analysis of all mass features from up to 77 Pinot noir red wines from Burgundy, spanning vintages from nearly all the 20th century down to 1915, and including the three SV1 samples (Fig. 3b). This analysis led to a straightforward remarkable differentiation of groups of wines along the first and the second axis (explaining 22.2 and 17.9% of the variance, respectively), with all of the wines produced after 1990, which appeared to be positively correlated to the first axis whereas wines older than 1990 were negatively correlated to the first axis (Fig. 3b). Several masses appeared to contribute to this age discrimination, with relative intensities either decreasing or increasing with the age of the wine (Supp. Fig. 5). From accessible databases or interfaces such as SciFinder Scholar or MassTrix,^27^ possible structural assignments for these specific masses could be polyphenols and in particular resveratrol, and residual sugars for masses, which decreased with age, and nitrogen containing compounds for masses which increased with age. However, almost 95% of discriminant masses could not be annotated, which is consistent with the actual lack of knowledge about the chemistry of wine ageing.^18^

## Discussion

Besides the question of the actual nature and characteristics of its contents, the discovery of this bottle has inevitably aroused the desire to identify its age. If we assume that this cellar was the first storage for the bottle, its contents should be younger than 1772—when the reconstruction of the abbey was achieved—but older than 1793, when the abbey was destroyed to ruins during the French Revolution (Supp. Fig. 1). However, the still preserved cellars were continuously used for wine storage from 1830 to about 1860.^28^ Therefore, the bottle could also have been buried during the first half of the 19th century. Further clues were provided by the radiocarbon analysis of the deposit, which suggested calibrated date ranges (95% probability) from either Cal AD 1680 to 1730 or Cal AD 1810 to 1930 (Supp. Fig. 1). Consequently excluding the first interval for historical reasons mentioned above, the wine should have been bottled after 1810 AD, a fact that implies that the bottle was buried during the 19th century. However, the contamination with the mixing of some amount of 21st century ‘post-bomb’ radiocarbon from the air with genuine ‘old’ radiocarbon in the wine (therefore older than 1810) cannot be excluded. In fact, this could have occurred between 2006—when the bottle was discovered and unearthed—and 2011, when the wine was finally sampled for the present analysis. In summary, the wine in the bottle could date either from the 1772 (or a few years before) to 1793 period, implying some post-bomb 21st century radiocarbon contamination; or, if no contamination occurred, from 1810 to 1860 (Supp. Fig. 1). Neither the typology of the bottle (from 1780 to 1820) nor archeo-historical data allowed us to choose between the first and the second period. Therefore the “old” wine analyzed in the present study was considered older than 100 years and potentially from the early 19th century, taking into account possible minimum and maximum ages of 150 and 239 years, respectively, when it was sampled in 2011.

Having answered to this historical question, the next step was to check the actual wine nature of the content of this bottle, although this was clear from its tasting. Thanks to high-resolution ^1^H NMR analyses (Supp. Fig. 2), relative concentrations of key wine markers such as ethanol, glycerol and tartrate could be measured (Table 1). Among interesting results, these analyses revealed in particular that the SV1 wine exhibited lower relative concentrations in ethanol and glycerol, and higher relative concentrations in methanol, tartrate, and acetate compared to the 1915 and 2007 wines. The lower concentration in ethanol could likely suggest either some evaporation during this long ageing (which would occur for conservation in moisture conditions), or an early harvest with lower ethanol potential. In contrast, the slightly higher methanol concentration would suggest a relatively higher content of pectins from the skins of grapes, which gave SV1. Higher concentrations of tartrate, lactate and acetate in SV1 also witnessed to a rather acidic wine (an early harvest would have low ethanol potential and high acidity due to high tartrate and malate levels), characterized by a malolactic fermentation which came to completion (high lactate), and with some volatile acidity (high acetate), the latter being sometimes associated positively with the ageing potential of a wine. A higher tartrate concentration also revealed that SV1 must have been kept in good temperature conditions (above 8–10 °C), minimizing any tartaric salt precipitation. However, from the lower glycerol concentration, combined with higher concentrations in lactate, acetate, and formate, it could also be considered that to some extent, an unwanted microbial degradation of glycerol may have occurred, leading to increased concentrations of the organic acids. Such degradation would also be consistent with the low gallic acid concentration (Table 1), since it produces acrolein, which interacts with tannins to contribute to some bitterness.^29^

Beyond such analyses of known metabolites, non-targeted FT-ICR-MS considerably widens the possible molecular description of wines by considering instantaneous description of all low mass metabolites than can be ionized under selected conditions.^19,22^ This is possible thanks to ultra high resolution, as exemplified by the signal at *m*/*z* 149.00915 (Supp. Fig. 6), corresponding to the [M − H]^−^ ion with [C_4_H_5_O_6_]^−^ absolute mass formula, assignable to tartaric acid, or by the signal at *m*/*z* 227.0713, corresponding to the [M − H]^−^ ion with [C_14_H_11_O_3_]^−^ absolute mass formula, most likely assignable to resveratrol isomers (Fig. 2a). Interestingly, if resveratrol was mostly present in recent wines (2007, 2008, and 2009), it was still detectable in the older wines, and in relatively higher concentrations in the deposit than in the wine, both for the 1915 and the SV1 wines (Fig. 2a). This is certainly the first time that resveratrol is shown to be preserved for such a long ageing.

The focus at *m*/*z* 227 nominal mass (Fig. 2a) provided a striking example of the chemical diversity that can be present in wines, and modulated by ageing, with up to 16 distinct *m*/*z* masses detected within a 400 mDa range (Supp. Fig.7). Out of the 16 corresponding elemental formulas, up to 12 could be identified in the RSV 1915 wine, whereas 10 and only 13 could be seen in the SV1 and RSV 2007 wines, respectively. This trend towards a modulation of the chemical diversity with ageing—and a decrease observed for very long ageing—was further illustrated both by the count of all detected common/specific masses of these wines in the Venn diagram (Fig. 2b) and by the count of some common compositional differences (Supp. Fig. 3). The former clearly showed that with ageing, both specific masses and masses common to young wines were decreasing. However, it also showed that when going from young to older wines, there was a progressive loss of CHO diversity in favor of N-containing compounds (CHON, Fig. 2c). The RSV 1915 wine thus appeared as an intermediate, still exhibiting few CHO specific masses (like young wines) but mostly N-containing ones (like SV1). It must be noted that this observation was consistent with ^1^H-^1^H TOCSY NMR spectra (Supp. Fig. 2), where the aromatic region characterized by condensed structures (which would be mostly CHO containing compounds) exhibited lower intensity in the older wines. This could be explained by precipitations of coloring matter (including tannins) occurring naturally over time during wine ageing for unfiltered and/or non-fined wines at bottling, which must have been the case most likely for the SV1 wine. However, common compositional differences further indicated that this modulation over time could be accompanied by various chemical transformations, which would be related to mechanisms such as hydration (H_2_O), *o*-methylation (CH_2_O) or sulfonation (SO_3_) (Supp. Fig. 3). The count of compositional differences thus showed that the number of compounds with elemental formulas differing by one o-methylation significantly decreased with ageing. In contrast, the number of N and NH_2_ transformations in SV1 samples—still comparable to younger wines—suggested that N-containing compounds, such as peptides—originally present in a young wine—could have been degraded into smaller amino acid-containing molecules, in agreement with specific masses identified for SV1 wines (Fig. 2c). It is also worth noting that the RSV 1915 wine exhibited more CH_2_ and OH compositional transformations than the young 2007 wine, thus emphasizing the time-related process of oenodiagenesis in bottle, which can proceed over periods as long as a century for wines with high ageing-potential. NH, S, or SO_3_ transformations appeared to be similar for the young 2007 wine and the nearly 100-year old 1915 wine (Supp. Fig. 3), which nicely relates to the ubiquitous nature of alkylsulfonic fatty acids, which could thus be involved in hundreds of chemical reactions throughout the wine ageing process. Altogether, this mass spectra analysis revealed great qualitative similarities between contemporary Romanée Saint Vivant wines, the 1915 and the SV1 wines, with comparable signal profiles in many nominal masses (Supp. Fig. 7), thus suggesting a likely filiation characterized by the fact that SV1 could have been made of Pinot noir grapes from the Côte de Nuits area, and possibly from the Saint Vivant vineyard in Vosne Romanée. This is particularly striking when the H/C vs. *m*/*z* van Krevelen diagrams of SV1 and RSV 1915 wines are compared (Fig. 2b).

Nonetheless, trying to identify both the grape variety and the age of an unknown old wine, based on its complex chemical fingerprint, remains challenging because of the lack of related references. In order to overcome this difficulty, we developed multivariate statistical analyses of all mass features—including those of SV1 samples—from a series of wines made from distinct grape variety (Fig. 3a), and from a vertical series of Pinot noir wines (Fig. 3b). Although only three grape varieties were considered in the Tonnellerie 2000 experiment^26^ (Fig. 3a), which prevents any definitve conclusion, SV1 wines appeared in the vicinity of Pinot noir wines, consistently with conclusions drawn above about the likely filiation with contemporary SV1 wines. But, the more striking result came from the straightforward PCA classification of SV1 samples among a remarkable series of up to 77 Pinot noir wines from Burgundy with vintages spanning nearly all the 20th century. SV1 samples readily appeared along a composite ageing axis (Fig. 3b) at the opposite of young RSV wines, and beyond the 1915 wine. SV1 samples exhibited the more negative correlation with this composite axis, thus confirming a vintage older than 1915 and potentially from the early 19th century. This group distribution along a composite ageing axis, clearly showed that the global SV1 wine matrix was progressively modulated by ageing, including through oenodiagenesis processes, but still holding chemical fingerprints remnant of a grape variety and/or a geographical origin.

Conclusions that could be drawn from this unprecedented report have been limited because of the small number of available old wines, which have been studied so far with metabolomics techniques. However, this archeochemical study is the very first attempt of identification of the grape variety of an old unknown bottled wine without genetic-based approaches. So far, only DNA extraction and amplification has shown efficiency to that respect.^30,31^ But, even if several studies have reported the ability to extract and genotype DNA from aged wine,^31–33^ efficient DNA extraction remains difficult^30,33^ due to winemaking practices, which significantly contribute to the removal of grapevine DNA from wine, and all the more after bottle ageing.^30,34^ Moreover, a major problem in acquiring information from archeological DNA is to prevent contamination from modern samples.^16,35^ Therefore, for the first time here the age of an unknown old wine along with its corresponding grape variety are assessed by non-targeted metabolomics analyses. Such result opens new perspectives for the authentication of wines, complementary to DNA-based methods.

## Methods

### Wine sampling

SV1 samples from the top, the middle and the bottom of the bottle, were collected immediately after opening the bottle (stored in the cellar of the Domaine de la Romanée Conti since its discovery). The wines were sampled into 2 ml vials under argon atmosphere to protect them from oxidation. For the purpose of age identification, 68 other Pinot noir red wines were collected from various vintages and appellations in Burgundy, including six Romanée Saint Vivant wines (1915, 2003, 2007, 2008 and 2009 vintages), five Pommard wines (vintage 1921), and 57 red wines from various appellations in Burgundy and various vintages between 1956 and 2009 (twenty six Côte de Beaune wines from 1976 to 2007, eight Grand Echezeaux and eight Richebourg from 1959, 1966, 1979, 1989, 1999, 2007, 2008, and 2009, five la Tâche from 1956, 1991, 2007, 2008, and 2009, four Romanée Conti from 1965, 2007, 2008, and 2009 and six Echezeaux from 1978, 1999, 2004, 2007, and 2008). For the purpose of variety identification, related FT-ICR-MS analyses realized on 36 wines from a previous experimental setup (Tonnellerie 2000^26^) were compared to SV1. In brief, the red wines from the Tonnellerie 2000 experiment were divided into three lots corresponding to the grape variety the wines were made of: Pinot noir (12 Mercurey wines), Syrah (12 Côte Rotie wines) and Grenache (12 Gigondas wines).^26^

### FT-ICR-MS acquisition

Wines were diluted in methanol (1/20) for Fourier Transform—Ion Cyclotron Resonance–Mass Spectrometry measurements. The SV1 bottom sample, mainly corresponding to amorphous precipitates was extracted in methanol, and the extract further diluted (1/20) for FT-ICR-MS. All samples were injected at a flow rate of 120 µL h^−1^.

Fourier Transform—Ion Cyclotron Resonance mass spectra were recorded on a solariX spectrometer (Bruker Daltonik, Bremen, Germany) equipped with a 12 Tesla superconducting magnet and an Apollo II electrospray ionization source. Ultra-high resolution of these spectra (*m*/*z* 100—1000; 4 megawords; 500 scans) is characterized by an exceptional resolving power >500,000. All spectra were externally calibrated on a methanol solution of arginine clusters (10 mg L^−1^), and further internally calibrated on a home-built list of recurrent wine compounds, including fatty acids, with linearity up to *m*/*z* 600, and with mass errors below 50 ppb.

### FT-ICR-MS spectra analysis

Very high accuracy in elemental formula assignments relies on the conjunction of an automated theoretical isotope pattern comparison with generated formulas validated by setting sensible chemical constraints (N rule; O/C ratio ≤ 1; H/C ratio ≤ 2n + 2; element counts: C ≤ 100, H ≤ 200, O ≤ 80, N ≤ 3, S ≤ 3 and P ≤ 1). FT-ICR mass spectra were exported to peak lists with a cut-off signal-to-noise ratio (S/N) of 4. Peak alignment was performed with maximum error thresholds of 1 p.p.m. and filtered for masses occurring in minimum of 10% of all samples.^22^

### Statistical analysis of FT-ICR-MS data

Mass filtering was performed in MS Excel 2010 (Microsoft, Redmond, USA), before statistical analyses run with Genedata Expressionist for MS 8.0 (Genedata, Basel, Switzerland), Simca-P 9.0 software (Umetrics, Sweden)^22^ and Past 3.05 (Øyvind Hammer, Natural History Museum, University of Oslo).^36^

### NMR spectra acquisition

An aliquot of 140 μL of each wine was mixed with 70 μL D_2_O buffer (100% D_2_O, 500 mM NaH_2_PO_4_/Na_2_HPO_4_, 0.1% TSP, pH 3.5) and transferred to 3 mm outer diameter NMR tubes (Hilgenberg GmbH, Malsfeld, Germany).

NMR spectra were acquired on a Bruker 800 MHz spectrometer (Bruker Biospin, Rheinstetten, Germany) operating at 800.35 MHz with a cryogenic QCI probe. 1D ^1^H spectra with the standard pulse sequence [recycle delay (RD)-90°-*t1*-90°-*tm*-90°-acquire FID] were acquired, with water suppression irradiation during RD of 2 s, mixing time (*tm*) set on 200 ms and a 90° pulse set to 8 μs. For each 1D spectrum, 1024 scans were recorded (64 K data points with a spectral width of 12 p.p.m.). 2D ^1^H-^1^H TOCSY spectra were acquired with phase-sensitive sensitivity-improved pulse with WATERGATE (3-9-19) and using DIPSI-2. For each 2D spectrum, 32 scans per increment were recorded (19228 × 1024 data points, acquisition time of 1 s, and 16 dummy scans). The spectral widths were set to 12 and 12 p.p.m. in the F2 and F1 dimensions, respectively.

### NMR spectra analysis

TopSpin 3.2 (Bruker BioSpin, Rheinstetten, Germany) software was used for spectra analysis. Free Induction Decays (FID) were line-broadened to 0.3 Hz (exponential decay multiplication) before Fourier transformation. Spectra were manually phased, baseline corrected and calibrated to TSP (*δ* 0.00), and imported to Matlab (Mathworks, Massachusetts, USA). Metabolite quantification was obtained through the measurement of the area under the curve (AUC) of the NMR peak from metabolites of interest, and stoichiometrically corrected by dividing through the number of the protons that give rise to the peak. The obtained value is an arbitrary unit.

## Electronic supplementary material

Supplemental material

## Electronic supplementary material

**Supplementary information** accompanies the paper on the *npj Science of Food* website (10.1038/s41538-017-0001-5).
