# Supplementary material for: Chemical messages from an ancient buried bottle: metabolomics for wine archeochemistry
Source: NPJ Sci Food. 2017 Oct 30;1:1. doi: 10.1038/s41538-017-0001-5 (PMC6548415; doi:10.1038/s41538-017-0001-5)
Supplement: Supplementary file 1 — Supplemental material [file 41538_2017_1_MOESM1_ESM.docx]

Chemical messages from an ancient buried bottle: metabolomics for wine archeochemistry.

Chloé Roullier-Gall^1,2^, Silke S. Heinzmann^2^, Jean-Pierre Garcia^3^, Philippe Schmitt-Kopplin ^1,2*^ and Régis D. Gougeon^4*^

**Supplementary information**


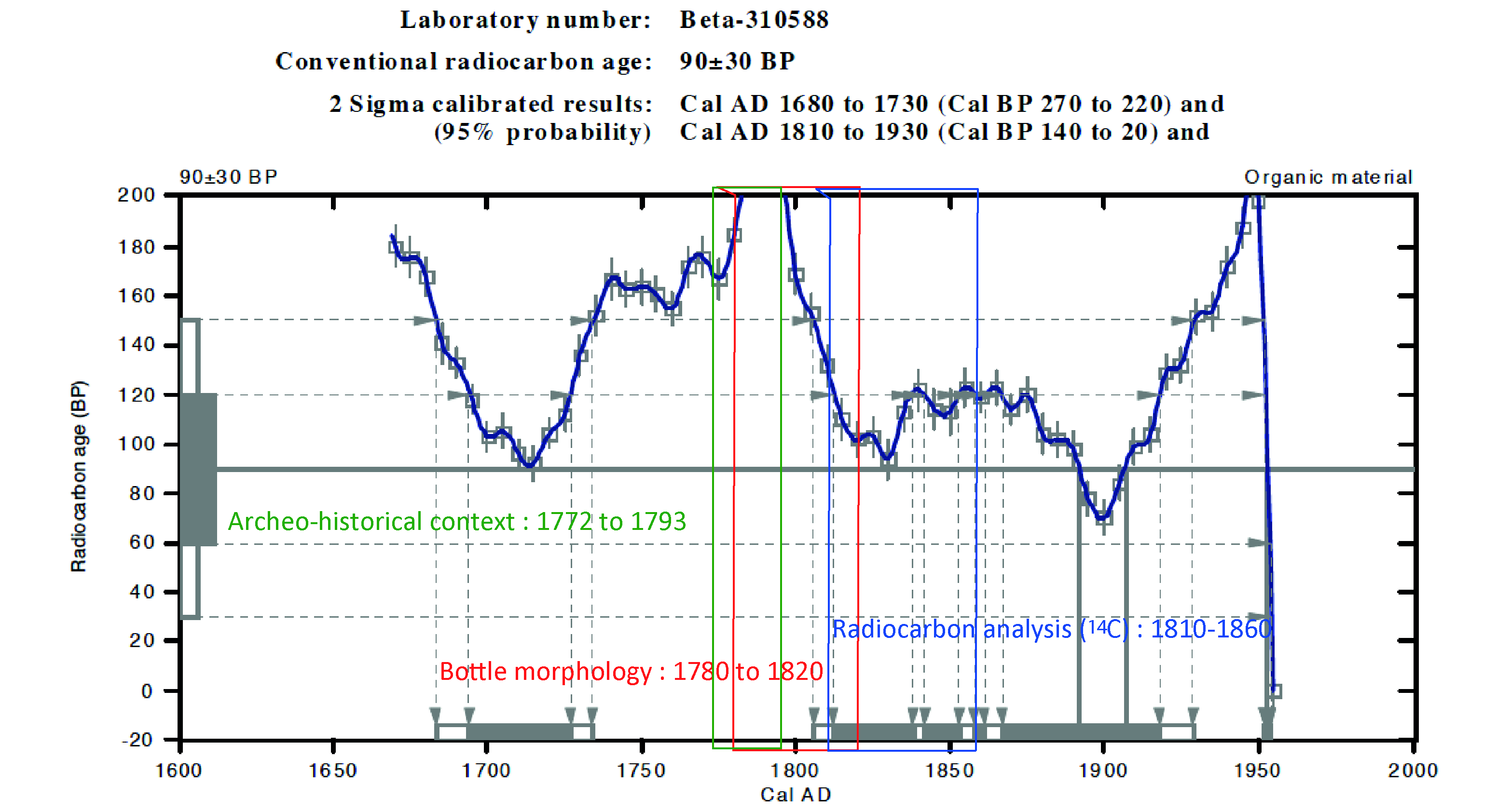


Supp. Figure 1: Estimation of the SV1 age according to archeo-historical context, bottle morphology and radiocarbon analysis (^14^C).

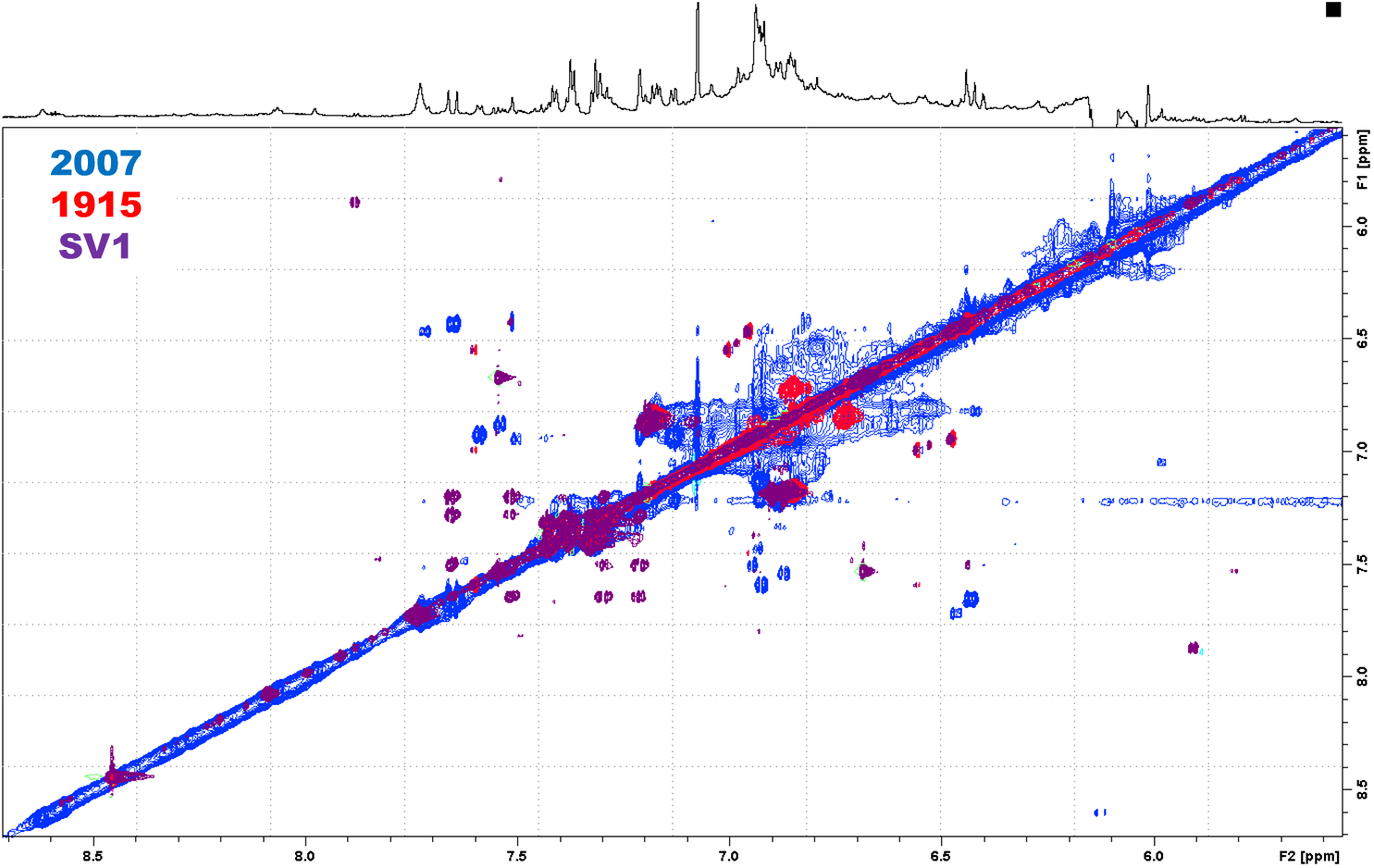


Supp. Figure 2: (Top) ^1^H NMR spectra of SV1 and RSV 1915, with focus on the aliphatic region (1 – 4.5 ppm) and the aromatic region (6 – 9.5 ppm). (Bottom) Overlap of ^1^H-^1^H TOCSY NMR spectra in the aromatic region (6 to 8.5 ppm) highlights the lack of dissolved macromolecular structures (broad background 6.5 – 7.2 ppm region) in the two old wines. Amino acids Tyrosine (6.9 ppm) and Phenylalanine (7.3 ppm) were present in similar amounts in all three wines. The polyphenol composition seemed different, as seen by the presence of different chemical shifts in the whole aromatic region.


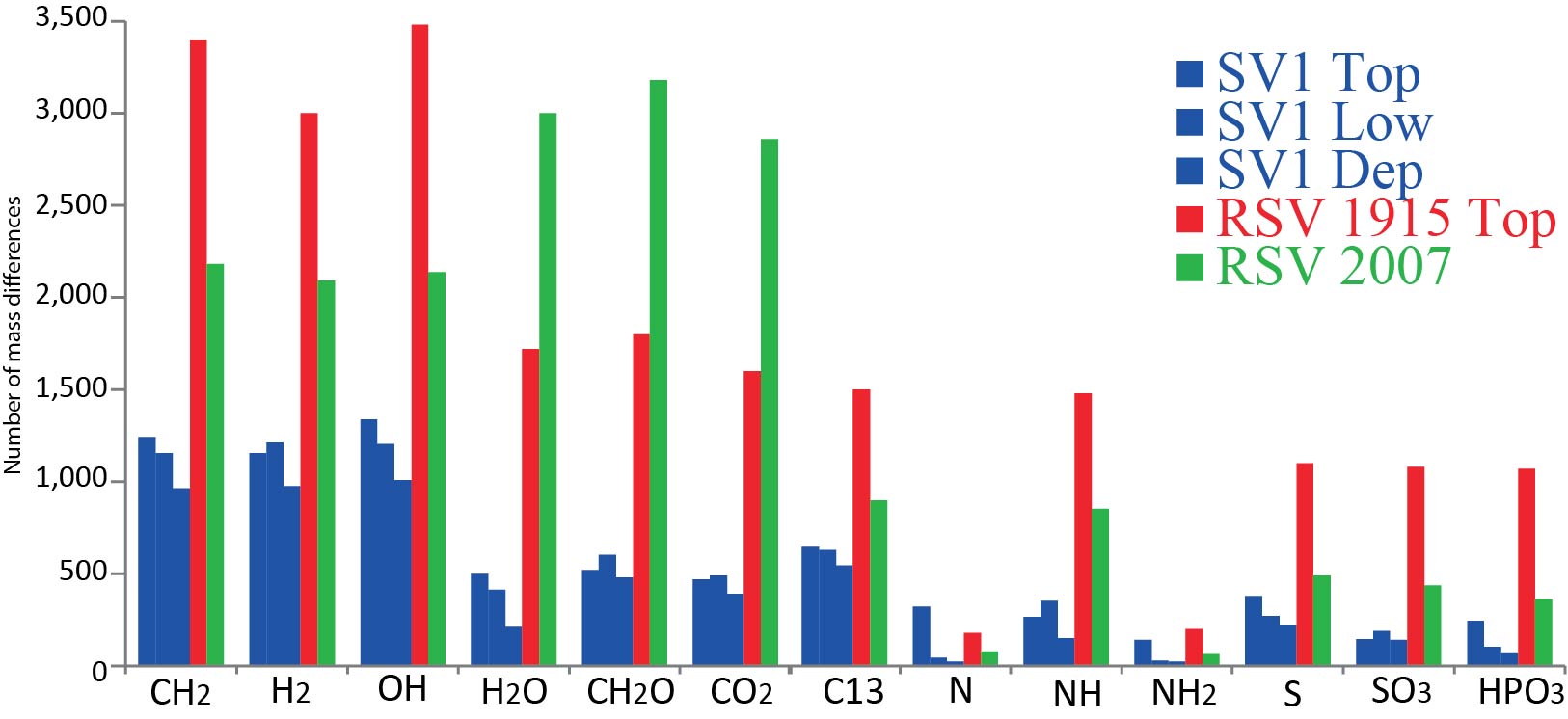


Supp. Figure 3: Frequency histogram of the number of exact masscompositionaldifferences calculated from all annotated masses fromSV1 (Top, Low and Dep), RSV 1915 and 2007 wines.


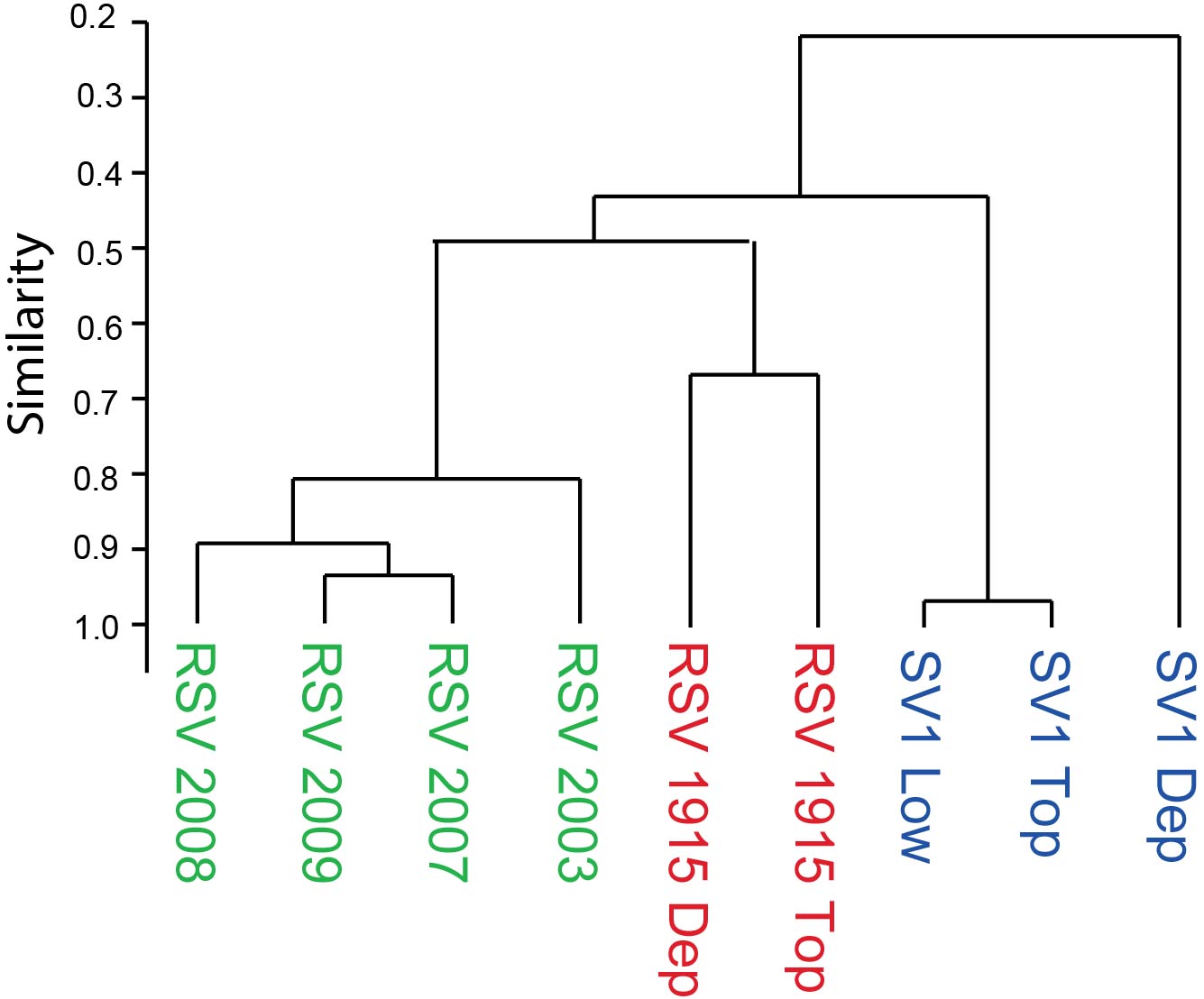


Supp. Figure 4: Hierarchical Cluster Analysis (HCA) of all mass features from wines from the three groups: RSV 2003, 2007, 2008, 2009; RSV 1915 (Top and Dep); SV1 (Top, Low and Dep).

Supp. Figure 5: H/C versus O/C van Krevelen diagram, with an example (m/z 323.13473 Da) of masses, which decreased with aging (A) and, which increased with ageing (m/z 181.05063 Da) (B). van Krevelen diagram color code: CHO, blue; CHOS, green; CHON, red; CHONS, orange).


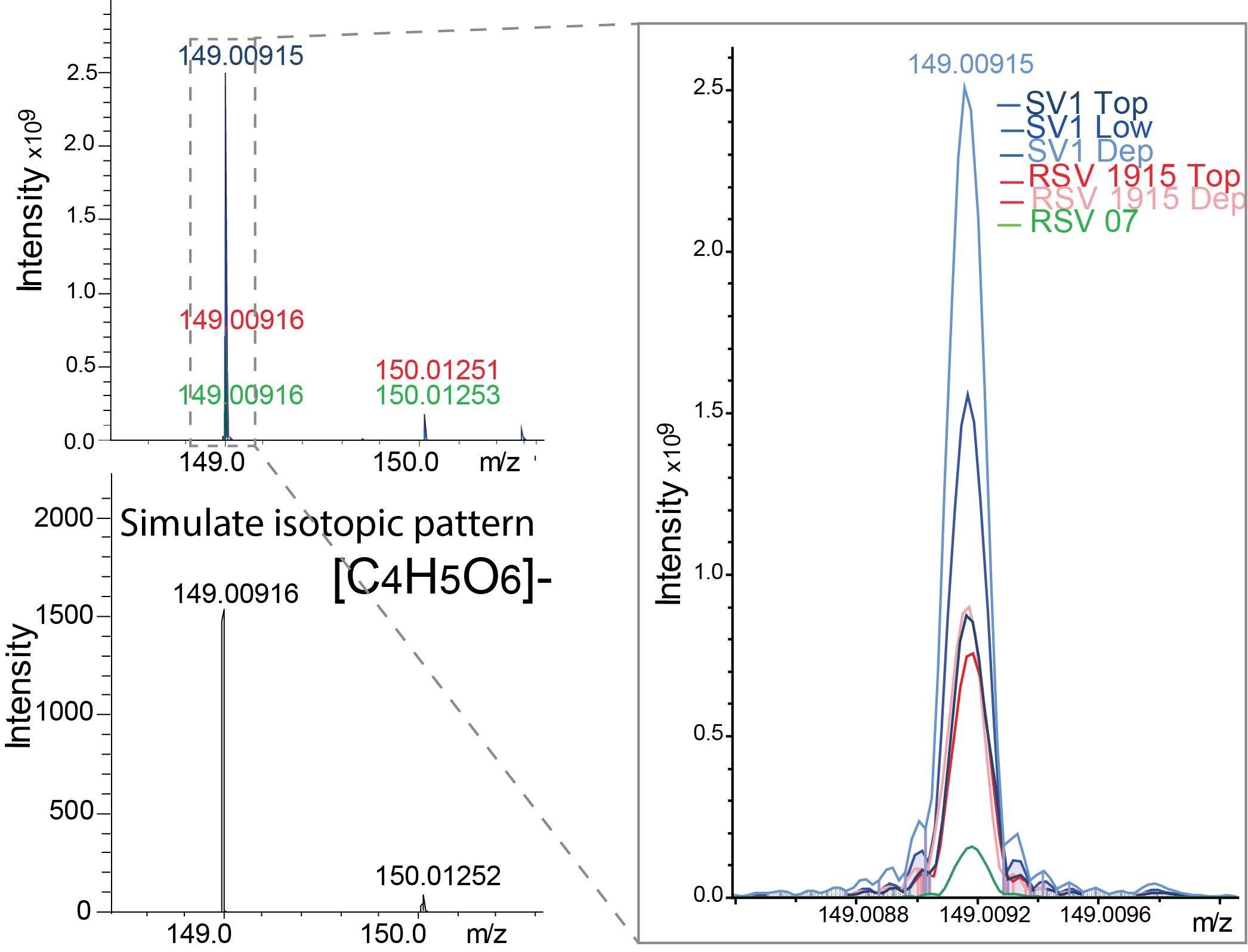


Supp. Figure 6: Tartaric acid (m/z = 149,00915 for the negative molecular ion), the principal biomarker for grape wine, was clearly detected in every sample, with a higher relative intensity in SV1 using FT-ICR-MS, in agreement with NMR results.


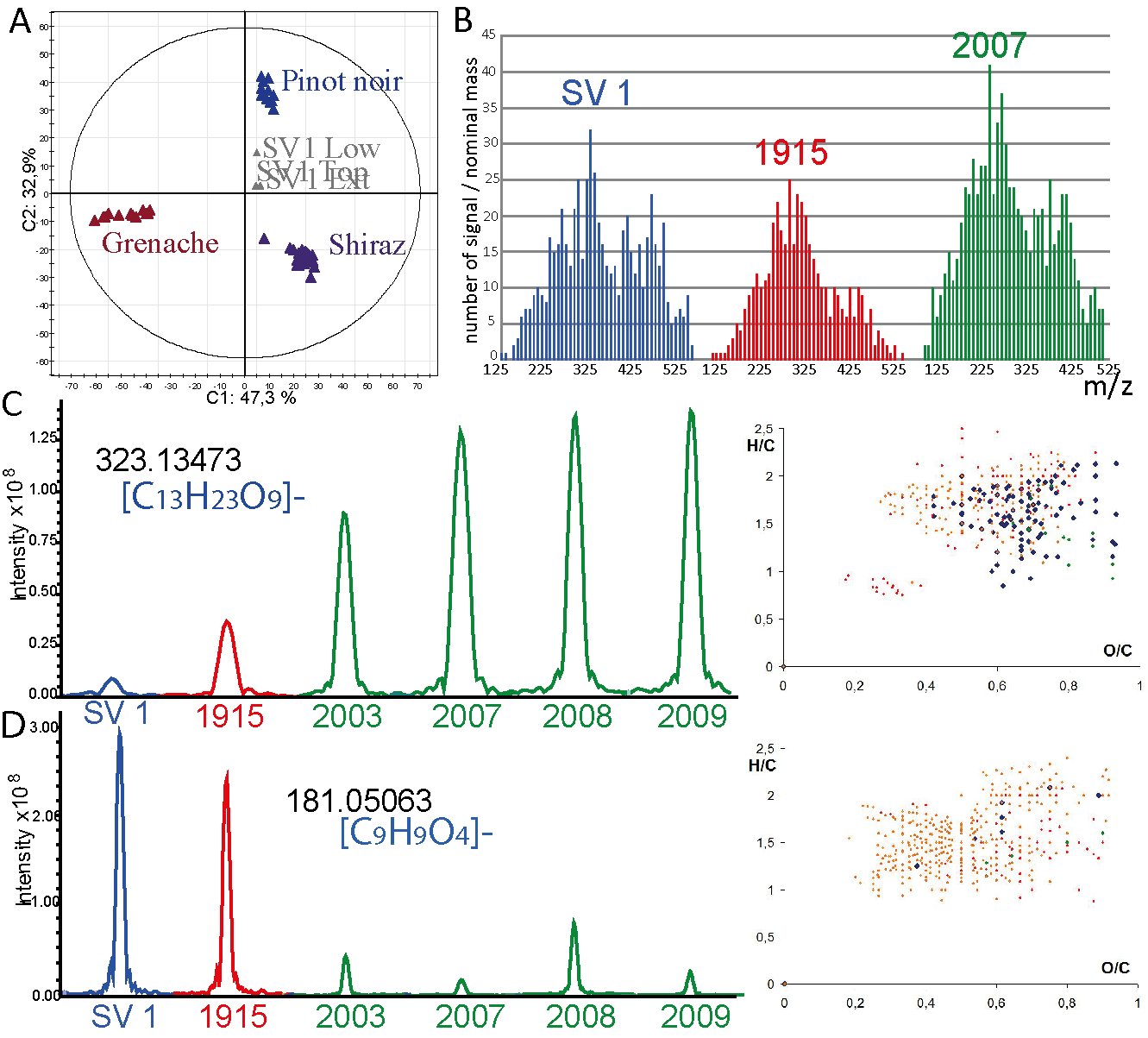


Supp. Figure 7: Histogram of number of annotated mass signals per nominal mass for SV1, RSV 1915 and RSV 2007.
